# Gaps between calcium recommendations to prevent pre-eclampsia and current intakes in one hospital in Argentina

**DOI:** 10.1186/1756-0500-7-920

**Published:** 2014-12-16

**Authors:** Gabriela Cormick, Nanci N Zhang, Simon P Andrade, María J Quiroga, Ingrid Di Marco, Andrés Porta, Fernando Althabe, José M Belizán

**Affiliations:** Department of Mother & Child Health Research, Institute for Clinical Effectiveness and Health Policy (IECS), Dr. Emilio Ravignani, 2024 Buenos Aires, Argentina; School of Public Health and Tropical Medicine, Tulane University, 1440 Canal St., Ste. 2430, New Orleans, LA 70112-2705 USA; Hospital Materno Infantil “Ramón Sardá”, Esteban de Luca 2151, 1246 Buenos Aires, Argentina; Laboratorio de Ingeniería Sanitaria, National University of La Plata, 47 N° 200, CP1900 La Plata, Argentina

**Keywords:** Perinatal, Prenatal, Healthcare, Pre-eclampsia, Argentina, Calcium intake, Maternal nutrition

## Abstract

**Background:**

Hypertensive disorders are a major cause of maternal mortality. In Latin America and the Caribbean, pre-eclampsia accounts for approximately one in every four maternal deaths. The World Health Organization recommends calcium supplementation during pregnancy for the prevention and treatment of pre-eclampsia and eclampsia in locations where dietary calcium intake is low. Calcium intake in Argentina is reported to be below WHO recommended levels; however, calcium intake from supplements and water has not been fully evaluated. The objective of this study was to evaluate calcium intake from supplements and water in a group of pregnant women.

**Methods:**

This cross-sectional study was conducted at a maternity hospital in the city of Buenos Aires, Argentina. Questionnaires were verbally administered to women attending a routine antenatal care visit. Participants were 18 years of age or older and in their third trimester of pregnancy. Participants were first interviewed to evaluate nutritional supplement consumption and a subgroup was invited to undergo a 24-hour dietary recall.

**Results:**

137 women meeting inclusion criteria consented to participate. The average participant age was 27 years (SD ± 5.9), and all resided in an urban setting. None of the subjects took calcium supplements specifically, although 24 (17%) recalled taking supplements or antacids which contributed to their calcium intake. Mean calcium intake was 663mg SD ±389 for those women completing the 24-hour dietary recall,. This value increased to 706 mg SD ±387 upon considering water intake and measuring chemical composition of water from the areas where women lived at the time of the interview and was further increased to 719 mg (SD ±392) when calcium from supplements was taken into consideration.

**Conclusions:**

None of the subjects were consuming calcium supplements. Taking into account the low calcium intake in this population, diverse strategies would be required to comply with recommendations.

## Background

Hypertensive disorders account for 18% of all maternal death world-wide with an estimated 62,000 to 77,000 deaths annually [1]. Pre-eclampsia, a gestational disorder defined as hypertension accompanied by proteinuria, is a major cause of maternal death in Latin America and the Caribbean, accounting for approximately one in every four maternal deaths [2, 3].

The hypothesis that increased calcium intake during pregnancy reduces the risk of pre-eclampsia was first postulated during the 80s [4, 5]. A systematic review of 13 randomised control trials shows that women who received calcium supplementation during pregnancy were less likely (RR: 0.45; 0.31- 0.65) to develop pre-eclampsia than women who received a placebo [2]. Further, a higher protective effect was observed among women with low basal calcium intake (less than 900 mg/day) (RR 0.36, IC 95% 0.20 a 0.65). In 2011, the World Health Organization (WHO) published recommendations for the prevention and treatment of pre-eclampsia and eclampsia, and classified calcium supplementation during pregnancy as a strong recommendation with moderate evidence in areas where dietary calcium is low. However there is some degree of controversy, as systematic review also shows a marginal increase of HELLP syndrome, the hypothesis is that as calcium supplementation reduces blood pressure and delays the diagnosis and treatment of pre-eclampsia, allowing more time for the condition to progress to HELLP syndrome [2, 6].

Calcium intake in Argentina is below recommended levels. The National Nutrition and Health Survey (ENNyS), a nationally representative survey carried out in 2005, estimated a mean intake of 529 (SD ± 389) mg/day in pregnant women using a single 24-hr recall [7]. Intake of calcium supplements was reported by only one percent of the women of fertile age, although consumption of calcium from water and multivitamin or mineral supplements was not assessed (ENNyS 2007). Another limitation of the ENNyS survey is that a single 24-hr recall was used and not the recommended multiple pass methodology that allows the several opportunities to verify the data reported [8].

The objective of this study was to evaluate calcium supplementation in a sample of pregnant women attending a leading maternity hospital in Argentina using a multiple pass 24-hour dietary recall questionnaire.

## Methods

This cross-sectional study of pregnant women was conducted during July and August 2012 at a maternity hospital in Buenos Aires, Argentina. The Hospital Materno Infantil Ramón Sardá is a comprehensive referral maternity hospital performing around 7,000 deliveries a year, mainly serving women living in both the capital of Buenos Aires (estimated population: 3 million inhabitants) and the surrounding Greater Buenos Aires area (estimated population: 10 million inhabitants) [9].

The healthcare system has 3 sectors: the labour union insurance funds, the public sector, and the private sector [10]. Women receiving care in public hospitals in Argentina typically are identified as belonging to a lower-middle socioeconomic class. They represent around 50% of the population, mainly by the 38% of the population who do not have formal work or cannot afford private insurance and by a fraction of those from the labour union insurance funds [9].

This study was approved by the Institutional Review Board (IRB) of Tulane University and the Ethics Committee of the Centre of Medical Education and Clinical Investigations in Buenos Aires, Argentina.

### Participants and procedures

Inclusion criteria required participants to be at least 18 years of age and in their third trimester of pregnancy (at least 27 weeks of pregnancy completed). The third trimester was selected to allow subjects’ recall of supplement intake during their current pregnancy.

Research personnel approached pregnant women in waiting rooms prior to the patients’ prenatal appointments and assessed their eligibility. Women were informed about the objectives of the study and those electing to participate were invited to sign the informed consent and then interviewed by the previously trained hospital dietician in a quiet nearby office. All women were first interviewed using a short questionnaire to obtain demographic characteristics including: maternal education, age and area of residence, and whether they were taking supplements or medicines known to contain calcium. The dietician was provided with a list of nutritional supplements and medicines containing calcium available in the market. Women were asked about frequency and dose of the supplements and medicines and if they were following professional recommendations. The type and dose of the supplements were recorded to develop a list of supplements most frequently consumed by pregnant women, and to assess supplement intake during pregnancy in this group. For this short questionnaire, sampling was performed by convenience. Interviewers recruited all eligible women in waiting rooms until completing the sample size.

A systematic sampling technique was used to evaluate dietary calcium intake. Every fifth woman taking the short questionnaire was asked to undergo a triple pass 24-hour dietary recall developed for this study to assess the participant’s nutritional intake in the previous day. After each 24-hour recall interview the dietician completed a face validity questionnaire, a subjective report evaluating if the interview recorded the food and drinks the women had consumed in the previous day.

### Calcium content of water

Women undergoing the 24-hour recall were specifically asked the type and amount of water consumed. Sources mentioned included commercially bottled water and tap water. Calcium content of commercially bottled water was obtained from nutrition information labels. Piped water, samples were obtained from the areas of residence that were reported by participants in the short questionnaire and were chemically analysed. Samples were collected following instructions of the Sanitary Engineering Laboratory of the University of La Plata.

Calcium content was determined using the ethylenediamine tetra acetic acid titration method (EDTA) that detects calcium from 2 mg/l of CaCO3. This method has a relative error of 1.9% and requires 25 ml of water of each sample [11].

### 24-hour recall questionnaire

The 24-hour recall was collected following the multiple pass methodology [12]. First, respondents were asked to report everything that they had to eat or drink on the previous day between midnight to midnight, then they were asked to report details of each food to obtain the amount consumed, and finally a review was done to prompt for items usually forgotten such as supplements, drinking water, snacks and sweets. Interviews were performed from Monday to Friday until the sample size was reached. As 24-hour recalls collect information on the previous day’s intake, we were able to register intakes of Sundays as the non-typical day.

Completed questionnaires were stored securely in binders by the interviewers and subsequently collected by research staff.

### Data analysis

Data was entered into Excel spreadsheets and measures of spread were calculated using SPSS Version 18.

The sample size of 135 subjects was estimated under the assumption that there were around 600 deliveries per month and that 10% of women took nutritional supplements with a +/- 4% error in the estimation, and a confidence level of 90%. A study conducted in the province of Buenos Aires showed that 19% of women interviewed after delivery reported taking vitamin or mineral supplements [13]. We estimated that 10% of women would be taking calcium supplements during the third trimester of pregnancy.

## Results

A total of 304 women were screened in the waiting rooms and 188 (61.8%) met the inclusion criteria, 172 (91.5%) of those eligible consented to participate while 16 (8.5%) refused. Of those enrolled, 137 (79.7%) women completed the interview, but 35 (20.3%) left before starting the interview.

Table 1 provides the demographic characteristics of women interviewed. The mean age of participants was 27 years (SD ± 5.9), all women reported living in urban areas, 29 (21.2%) had 7 or less years of formal education, 123 (89.8%) of them did not have a health provider and 54 (39.4%) were nulliparous.

**Table 1:** **Demographic information**

		All woman	Dietary assessment subgroup
**Characteristics**		(n = 137)	(n = 29)
		n (%)	n (%)
Age (years)	Less than 20	13 (9.5)	7 (24.1)
	20 to 34	109 (80.0)	20 (69.0)
	35 and more	15 (11.0)	2 (6.9)
Country of birth	Argentina	69 (50.4)	19 (65.5)
	Bolivia	36 (26.3)	5 (17.2)
	Paraguay	18 (13.1)	3 (10.3)
	Peru	12 (8.8)	2 (6.9)
	Other	2 (1.4)	0 (0)
Area of residence			
	City of Buenos Aires	59 (43.1)	11 (37.9)
	Province of Buenos Aires	78 (56.9)	18 (62.1)
Health provider	Labour Union	14 (10.2)	4 (13.8)
	None	123 (89.8)	25 (86.2)
Number of children	Nulliparous	54 (39.4)	16 (55.2)
	Multiparas	79 (57.7)	13 (44.8)
	Missing	4 (2.9)	0 (0)
Year in formal education	7 years or less	29 (21.2)	8 (27.6)
	More than 7 to less than 12 years	37 (27.0)	6 (20.7)
	12 years	35 (25.5)	6 (20.7)
	More than 12 years	36 (26.3)	9 (30.1)

None of the 137 women were specifically consuming calcium supplements, however 24 (17%) were taking a supplement or a medication containing between 100 and 320 mg of elemental calcium. 14 (10%) reported taking supplements containing calcium, 9 (7%) reported taking antacids and 1 reported taking both composite supplements and antacids. The supplements or medicines provided an average of 148 mg per day for those 24 participants that consumed any of them and 25 mg per day when averaging the amount within the 137 participants.

### Calcium content of water

All 137 women taking the short survey lived in areas supplied by water networks, however the coverage varies and can be divided in three main sectors; the city of Buenos Aires where 99% of the population is covered by water networks, the Greater Buenos Aires areas with at least 80% of the population is covered by water networks, and other Greater Buenos Aires areas with around 50% is covered by water networks. The areas not supplied by water networks are served by local wells.

Fifty-nine (43.1%) women resided in the city of Buenos Aires, 68 (49.6%) came from Greater Buenos Aires with 80% coverage and 9 (6.6%) women lived in areas where water networks cover around half of the population. Eleven samples of water were analysed, 8 (72.7) samples came from areas mainly covered by water network supplies and 3 samples came from wells.

Tap water from network supplies contained an average of 34 mg of calcium per litre with a range from 25 to 88 mg/l and that from local wells 100 mg of calcium per litre with a range from 28 to 173 mg/l. Bottled mineral water contained an average of 38 mg/l of calcium with a range from 25 to 78 mg/l.

Of the 29 women undergoing the 24-hour recall 13 (44.8) came from areas with 80% coverage, 11 (37.8%) came from the city of Buenos Aires and 5 (17.2%) from areas with around 50% coverage.

### 24 hour recall questionnaire

The 24-hour dietary recall was administered to 29 women. The questionnaire administration took approximately 18 minutes to complete (range 9–35 minutes). Women’s mean age was 24.7 years (SD ± 6.03), with 8 individuals (27.6%) reporting highest education level of primary school or less and 16 (55.2%) were nulliparous.

Of these women that underwent the 24-hour recall, 7 (24%) were interviewed on a Monday, thus reporting their Sunday intakes, which represents a non-typical day. The rest of the interviews were on weekdays and the day distribution was 3 (10%) on Tuesdays, 5 (17%) on Wednesdays, 10 (34%) on Thursdays and 4 (14%) on Friday.

Mean energy intake was 1874 (SD ±487) kcal composed of 246 (SD ±76) (52%) grams of carbohydrates, 63 (SD ±20) (14%) grams of protein, and 71 (SD ± 27) (34%) grams of fat (Table 2).

**Table 2:** **Energy, protein, fats, carbohydrates and calcium intake**

	n	Minimum	Maximum	Mean	Std. deviation
Energy (Kcal)	29	844.7	2967.3	1874.2	487.4
Protein (grams)	29	37.8	111.0	63.3	19.8
Lipids (grams)	29	22.4	120.5	71.0	27.0
Carbohydrates (grams)	29	78.0	415.5	245.7	76.5
Calcium (miligrams)	29	144.6	1679.3	706.5	386.7

From the 29 women that finished the 24-hour recall, 9 (31%) reported taking a calcium containing supplement or medication which provided an average of 134 mg of calcium per day and 42 mg if the amount was averaged by all 29 participants.

All 29 women reported drinking tap water and 13 mineral water as well. As it is difficult to assess whether the tap water consumed came from a water networks or a well, we calculated total calcium intake from tap water using mean values of water network supply only, as this type of water is available in all areas. The exact chemical composition of bottled water was used when participants provided details of the brand, otherwise the average was used. Total water intake provided in average 43 mg of calcium a day (SD ± 27).

Mean calcium intake was 663 mg (SD ±389). Mean calcium intake increased to 706 mg (SD ±387) when calcium from water intake was included and to 719 mg (SD ±392) when calcium from supplements was considered.

Compared to what the women recalled as their usual diet, 17 reported that the amount consumed was similar to other days, 9 reported it was less and 3 that it was more than usual. Reasons for eating less included: not hungry (2), dieting (2), little food at home (1), busy (1), special occasion (1) and no reason (2). Those reporting having more than usual mentioned that the previous day was a weekend or that they had cravings. Women were also asked to compare the food reported with that prior to pregnancy; 10 women reported having a similar intake, 10 reported higher consumption and 9 reported lower consumption.

To assess the validity of the recall questionnaire, the dietician administering the interviews qualified 27 out of 29 24-hour recalls as good in the way they represented women intakes, whereas 2 were qualified as poor or moderate.

## Discussion

This study shows that, in a maternity hospital in Buenos Aires, there is no compliance with taking the 1,5 grams of calcium recommended by WHO guidelines for the prevention of pre-eclampsia [6]. None of the subjects took calcium supplements specifically, although 24 (17%) of them took multivitamin supplements or antacids containing between 100 and 320 mg of elemental calcium. We also found that in a sub-sample of this study calcium intake is below recommendations.

One advantage of this study is that evaluates calcium from supplements, medicines and water and those values were taken into account when calculating total calcium intake.

The assessment tool used in this study was a triple-pass 24-hour dietary recall, a fairly common technique among nutritional studies. It is subject to less recall bias than diet histories and food frequency checklists, which rely upon a greater recall period [14, 15]. Further, the 24-recall method does not require high literacy of the respondent and minimises inter-observer differences [16]. However a single 24-hour recall does not allow estimating usual calcium intake or the percentage of inadequate nutrient intake within a population, as these values are not adjusted for within-person variability [14].

One limitation of this study is that almost 19% of women left before starting the interview and 8% refused to participate. A small number of women were interviewed for the dietary assessment and this limits the conclusion about total calcium intake in this population.

The information in this study was similar to that shown in the ENNyS where only 1% of women of child-bearing age were taking calcium supplements, although calcium supplement intake of pregnant women was not reported [7].

Although the amount of calcium in water is relatively low, it is an interesting source of calcium considering that water intakes are high and calcium water has a good bioavailability, similar to that of cow’s milk [17]. The samples analysed in this study are representative for those areas supplied by the public water network. Further analysis is required to assess areas with wells, as calcium levels can vary significantly depending on the area. According to the samples analysed in this study, water from public networks seems to have lower calcium content than water from wells. The total calcium provided by water intake could be higher for this population, as the water network chemical composition was used for the analysis of calcium intake.

In a sub-sample of this study we found that calcium intake is below recommendations, however the value is higher than that reported by the ENNyS in 2007 [7]. This could be explained by an improvement of dietary calcium intake in the population or by the fact that women in the sub-sample were younger; the same ENNyS shows a higher intake in non-pregnant women younger than 19 years. More women in this sub-sample also consumed supplements or antacids containing calcium. This might show a different behaviour that can reflect also a difference in food patterns.

While information provided in this study is not strictly representative of the population in Argentina, women receiving care in public hospitals in Argentina represent around half of the population, 38% of those population who do not have formal work or cannot afford private insurance and a fraction of those from the labour union insurance funds [9]. Also there is some evidence that calcium intake is low in the country and represents a nutritional problem [7]. Furthermore, a recent review of worldwide studies reporting dietary intakes of pregnant women from low and middle-income countries show consistent low calcium intakes across Asia, Africa and Latin American countries [18]. In Latin America, only Mexico and Ecuador were above the 833 mg a day Estimated Average Requirement [19].

Despite being known for many years that adequate calcium intake prevents preeclampsia, calcium intake in this population is still low. This study shows that this group of pregnant women does not use supplements to improve their calcium intake as it is recommended by the recent WHO Guidelines in order to reduce the risk of having preeclampsia [6]. Similar results were shown in one large study carried out in Brazil, where 788 women with also low calcium intakes were interviewed and less than 6% of women received the prescription for calcium supplements [20].

We did not assess whether health providers at this hospital are taking action to improve maternal calcium intake such as prescribing calcium supplements or giving dietary advice. However, it seems that in low and middle-income countries there is poor adherence of women to nutritional supplements such as calcium, folic acid or iron [18, 21, 22]. Therefore, strategies to increase calcium intake should be explored and developed to improve calcium intake in this population.

## Conclusions

None of the subjects were consuming calcium supplements. Taking into account the low calcium intake in this population, diverse strategies would be required to comply with recommendations.

## Abbreviations

ENNyS: Encuesta Nacional de Nutrición y Salud. National Nutrition and Health Survey
WHO: World Health Organization.
